# High level expression of AMAP1 protein correlates with poor prognosis and survival after surgery of head and neck squamous cell carcinoma patients

**DOI:** 10.1186/1478-811X-12-17

**Published:** 2014-03-12

**Authors:** Hiroki Sato, Kanako C Hatanaka, Yutaka Hatanaka, Hiromitsu Hatakeyama, Ari Hashimoto, Yoshihiro Matsuno, Satoshi Fukuda, Hisataka Sabe

**Affiliations:** 1 Department of Otolaryngology, Head and Neck Surgery, Hokkaido University Graduate School of Medicine, W15N7 Kitaku, Sapporo 060-838, Japan; 2 Department of Surgical Pathology, Hokkaido University Hospital, W15N7 Kitaku, Sapporo 060-838, Japan; 3 Department of Molecular Biology, Hokkaido University Graduate School of Medicine, W15N7 Kitaku, Sapporo 060-838, Japan

**Keywords:** HNSCCs, AMAP1, EGFR, Overall survival, Disease free survival

## Abstract

**Background:**

Despite recent advances in cancer therapeutics in general, the survival of patients with head and neck squamous cell carcinomas (HNSCCs) has not improved substantially over the past few decades. HNSCC cells often exhibit invasive and metastatic phenotypes, and expression of epidermal growth factor receptor (EGFR) and cortactin has been highly implicated in the development of malignancy in HNSCCs. We have shown previously that an Arf6 pathway, in which Arf6 is activated by GEP100 and employs AMAP1 (also called DDEF1 or ASAP1) as its downstream effector, is pivotal for the invasion and metastasis of different breast cancer cells. This pathway is activated by receptor tyrosine kinases, including EGFR; and moreover, AMAP1 physically associates with cortactin, in which inhibition of this binding effectively blocks invasion and metastasis. We here investigated whether the expression of Arf6 pathway components correlates with the poor prognosis of HNSCC patients. We have shown previously that AMAP1 protein levels are not correlated with its mRNA levels, and hence we here employed immunohistochemical staining of HNSCC clinical specimens to investigate AMAP1 protein levels.

**Results:**

We found that high levels of AMAP1 protein expression on its own, as well as its co-overexpression with EGFR statistically correlates with poor disease-free survival and poor overall survival, while high levels of cortactin expression or its co-expression with EGFR did not.

**Conclusion:**

Our identification of predictive biomarkers, together with our previous findings on the coherent signaling pathway that these biomarkers ultimately generate should be powerful information for the further development of HNSCC therapeutics.

## Background

Head and neck squamous cell carcinoma (HNSCC) is the sixth most common cancer in the world [1]. There are about 500,000 new HNSCC cases reported annually worldwide [1]. Molecular studies have shown that aggressive HNSCCs frequently have mutations in the *TP53* gene, and show high levels of expression of cyclin D1 and epidermal growth factor receptor (EGFR) [2-6]. EGFR and its signaling, as well as high expression levels of cortactin, have been highly implicated in the aggressiveness of HNSCCs and the poor prognosis of patients [7-10]. Accordingly, Cetuximab, a monoclonal antibody against EGFR, has been commonly used to treat recurrent HNSCCs, by itself or in combination with platinum-based cytotoxic agents [11,12]. However, the therapeutic effects of Cetuximab are not uniform, and moreover, most recurrent HNSCCs eventually acquire resistance to such treatments using antibodies and chemicals. Other types of cancers like lung cancer often bear mutations within the *EGFR* gene [13-15], and colon cancer often exhibits mutations in the *k-ras* gene, which acts downstream of EGFR. These mutations may evoke resistance to the EGFR-based agents [16]. However, mutations in the *EGFR* and *k-ras* genes have been shown to be rare in HNSCCs [17]. Recent large-scale of the exome-analyses using next-generation sequencers have also revealed rare alterations in these genes in HNSCCs [18]. Moreover, with regard to EGFR and its signaling, previous studies by other research groups have primarily focused on the classical components, such as Ras, Raf, Erks, phosphatidylinositol 3-kinase, Akt and STAT-3 [19].

We have identified previously another signaling pathway under EGFR, which mediates invasive and motile phenotypes of cancer cells. We have shown that an Arf6 pathway, in which Arf6 is activated by GEP100, a guanine nucleotide exchanging factor (GEF) for Arf-GTPases, and employs AMAP1 (also called DDEF1 or ASAP1) as its downstream effector, is pivotal for the invasion and metastasis of different breast cancer cells [20-25]. In this Arf6 pathway, GEP100, via its PH domain, physically associates with tyrosine phosphorylated EGFR to activate Arf6 at the plasma membrane [23]. Both the Arf6 and AMAP1 proteins are abnormally overexpressed in highly-invasive breast cancer cell lines, while their expression is minimal in weakly- and non-invasive breast cancer cell lines and also in a primary culture of normal mammary epithelial cells [20,21]. Therefore, this Arf6 pathway appears to be specific to some cancer cells, and may not normally be used in normal cells. Studies on clinical specimens of breast cancer revealed that high expression levels of the AMAP1 protein as well as the co-overexpression of GEP100 with EGFR correlate well with their malignant and invasive phenotypes [21,23]. We have moreover shown previously that expression levels of the Arf6 and AMAP1 proteins do not correlate with their mRNA levels [20,21], suggesting that the over-expression of these proteins are not simply a result of the over-expression of their mRNAs. This may be the major reason why these proteins and their genes have not been identified to correlate with the invasive and malignant phenotypes of breast cancers by previous gene expression profiling analyses.

As for the molecular mechanisms of the Arf6 pathway in invasion, we have shown previously that activation of Arf6 by GEP100, but not by other GEFs, perturbs E-cadherin-based cell-cell adhesion of breast cancer cells and may hence induce their motile phenotypes [23]. On the other hand, AMAP1 has multiple protein-interacting modules and can directly interact with different proteins, including protein kinase D2 (PRKD2) and cortactin. AMAP1, via its binding to PRKD2, makes a complex with β1 integrins, in order to mediate the recycling of β1 integrins, leading to cell invasion [24]. Interaction of AMAP1 with cortactin is also crucial for invasion and metastasis, in which we have shown that blockage of this binding by a cell-permeable peptide, namely P4-TAT, effectively blocks breast cancer invasion *in vitro* and metastasis *in vivo*[22].

We here investigated whether protein expression of Arf6 pathway components, as well as EGFR and cortactin, exhibits clinical relevance to the malignant phenotypes of HNSCCs, with the aim to investigate whether components of the Arf6 pathway could be predictive biomarkers and therapeutic targets.

## Results

### High expression levels of AMAP1 correlate with poor prognosis

To investigate whether the increased expression of certain proteins is associated with disease-free survival as well as overall survival of HNSCC patients after curative resection of the primary sites, we classified patients (Table 1) into two groups based on the results of immunohistochemical stainings.

**Table 1:** Clinicopathologic and characteristics of patients

**Characteristic**	**No. of patients (%)**
Sex	
Male	15/20 (75)
Female	5/20 (25)
Age	
30—39	3/20 (15)
40—49	2/20 (10)
50—59	4/20 (20)
60—69	6/20 (30)
70-	4/20 (20)
Location	
Oral cavity	20/20 (100)
T classification	
T1	0(0)
T2	13/20 (65)
T3	7/20 (35)
T4	0(0)
N classification	
N0	10/20 (50)
N1	7/20 (35)
N2	3/20 (15)
Differentiation	
Well differentiated	2/20 (60)
Moderately differentiated	7/20 (35)
Poorly differentiated	1/20 (5)
Radiation therapy	
Yes	9/20 (45)
No	11/20 (55)
Chemotherapy	
Yes	3/20 (15)
No	17/20 (85)

Clinical specimens were selected from patients undergoing surgical resection for head and neck squamous cell carcinoma. The tumor site was the oral cavity (tongue) in all patients.

**Figure 1:**
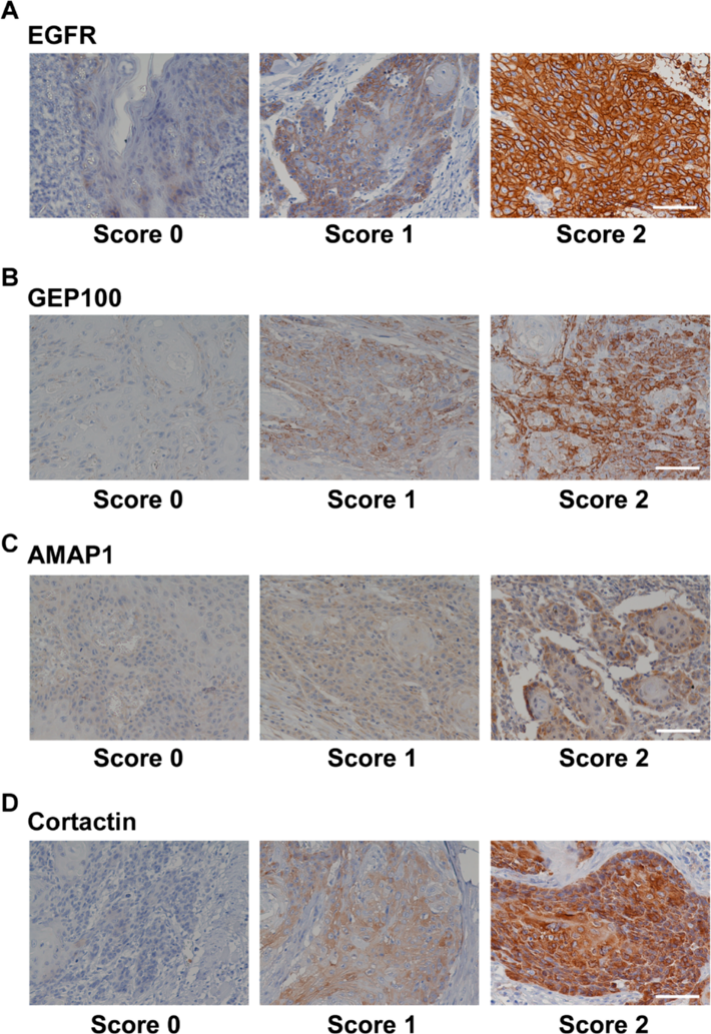
**Immunohistochemical staining of EGFR (A), GEP100 (B), AMAP1 (C), and cortactin (D) in primary HNSCCs.** Tissue sections were immunostained with antibodies against each target protein, as indicated. Positive staining of the proteins is shown in a reddish-brown color. The staining intensity of each protein in tumor cells was graded on a scale of 0–2, as described under *Materials and methods.* A representative figure for each staining is shown. Bars, 100 μm.

The median H scores for HNSCCs were found to be 0.65 (EGFR), 0.6 (GEP100), 0.3 (AMAP1) and 0.3 (cortactin) (Figure 1A-D, also see *Materials and methods*).

**Figure 2:**
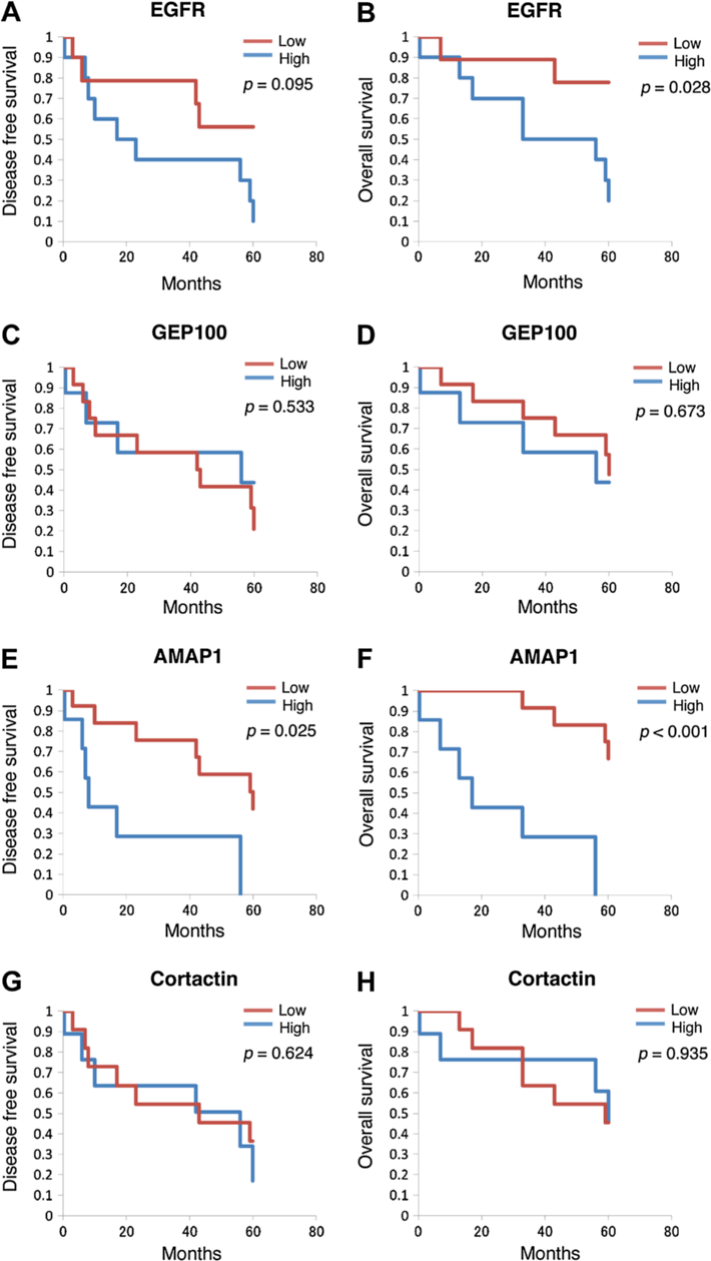
**Kaplan-Meier curves for the 5-year disease-free survival and overall survival of patients with HNSCCs expressing each single protein.** Patients were subclassified into the high-expression group and the low-expression group based on results of the immunostainings, as described under *Materials and methods.* Kaplan-Meier curves were drawn with regard to the disease-free survival and the overall survival **(A, C, E, and G)** of patients **(B, D, F and H)**, by comparing the high-expression group and the low-expression group of each target protein, as indicated.

Tumors with scores above the median H value were classified into the high expression group, and those below the median H value were classified into the low expression group. We found that the 60-month disease-free survival for patients in the high AMAP1 expression group is 0%, while it is 42% for those in the low AMAP1 expression group (*p* = 0.025, Figure 2E). On the other hand, there were no significant differences in expression of EGFR, GEP100 or cortactin with regard to the 60-month disease-free survival (Figure 2A, C and G).

We next analyzed the overall survival of patients. The 60-month overall survival of patients in the high AMAP1 expression group was 0%, while it was 67% for patients in the low AMAP1 expression group (*p* < 0.001, Figure 2F). The 60-month overall survival of the patients in the high EGFR expression group was 20%, while it was 78% for patients in the low EGFR expression group (*p* = 0.028, Figure 2B). There were no significant differences in survival rates between groups with different expression levels of GEP100 as well as groups with different expression levels of cortactin (Figure 2D and H).

### Co-expression of EGFR and AMAP1 both at high levels correlates with poor prognosis

**Figure 3:**
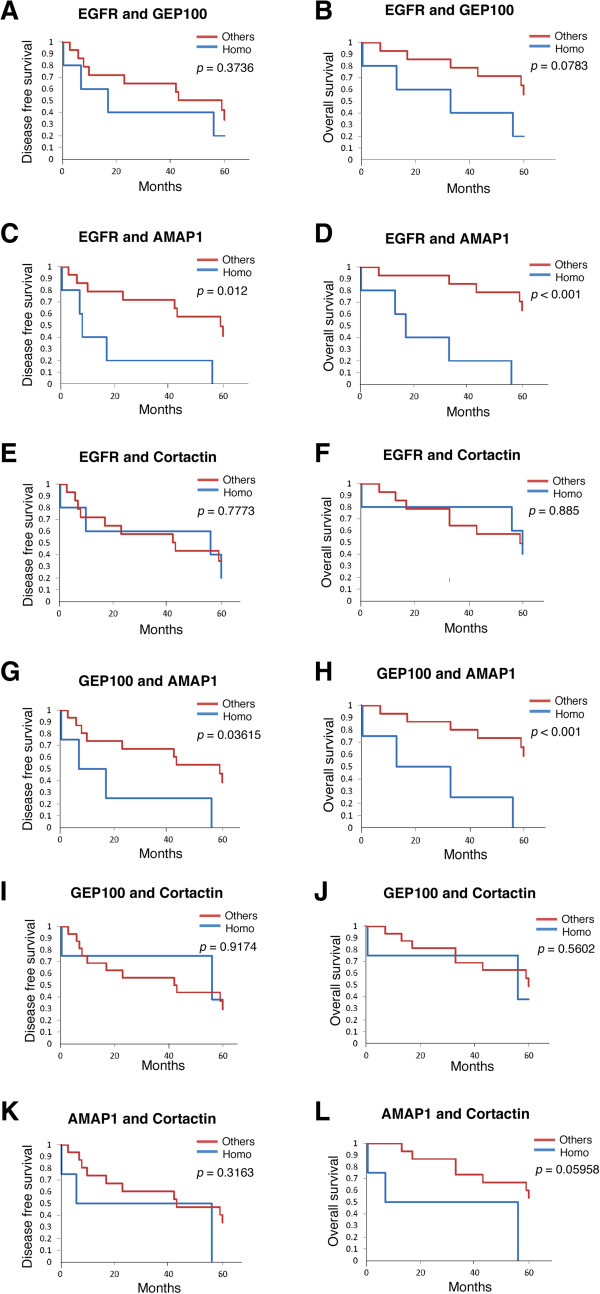
**Kaplan-Meier curves for the 5-year disease-free survival and overall survival of patients with HNSCCs expressing a combination of each of the two different proteins.** Patients were subclassified into the 'Homo' group and the 'Others'. The Homo group consists of those expressing both target proteins at high levels (*i.e.*, both belong to the high-expression groups), while Others consist of the rest of them. Kaplan-Meier curves were drawn with regard to the disease-free survival **(A, C, E, G and I)** and the overall survival of patients **(B, D, F, H, J and L)**, by comparing the Homo group vs. the Others.

We then analyzed whether high-level expression of a combination of these proteins exhibits greater differences in disease-free survival and overall survival compared with the high expression of each single protein. Due to the relatively small number of clinical samples in our hospital, we could only analyze combinations of two different proteins with statistical significance, but not the combinations of more than three proteins. We found that the EGFR/AMAP1 'Homo' group, in which expressions of EGFR and AMAP1 both belong to the high-expression groups, shows a shorter time to events after HNSCC resection than the 'Others', in which either one of the AMAP1 expression or the EGFR expression, or both belong to the low-expression groups (*p* = 0.012 for the disease-free survival and *p* < 0.001 for the overall survival, Figure 3C and D). This difference in the disease-free survival was greater than that for EGFR alone and AMAP1 alone, and the difference in the overall survival was greater than that for EGFR alone. The GEP100/AMAP1 'Homo' group, in which expressions of GEP100 and AMAP1 both belong to the high-expression groups, also shows a statistically shorter time to events after HNSCC resection than the 'Others', in which either one of the GEP100 expression or the AMAP1 expression, or both belong to the low-expression groups (*p* = 0.03615 for the disease-free survival and *p* < 0.001 for the overall survival, Figure 3G and H). This difference was, however, not greater than that for AMAP1 alone. We also examined other combinations, and found that none of them exhibit a statistical difference with regard to disease-free survival and overall survival (Figure 3A, B, E, F, I-L).

## Discussion

Despite the recent advances in cancer therapeutics, the overall survival of HNSCC patients after the curative resection of tumors has not improved significantly over the past few decades [26,27]. Among the many different factors that contribute to poor survival and poor prognosis, the major cause is believed to be the invasive and metastatic properties of some HNSCCs, which are still far from under clinical control. Interestingly, HNSCCs show the highest frequency of high EGFR expression levels among all different types of cancers, and hence EGFR-targeted therapies are expected to exhibit beneficial effects [6-8]. Indeed, combination of Cetuximab with platinum-based chemotherapies, as well as with conventional radiotherapy have been shown to improve the disease-free survival rates of HNSCC patients, while these therapies still do not significantly improve their overall survival [9,12,28]. Inhibition of the downstream signaling components of EGFR, such as Erks and Akt, was also thought to be a candidate to block tumor growth, but this greatly affects the survival and functions of most normal cells and hence often evokes severe side effects [19].

The EGFR-GEP100-Arf6-AMAP1 pathway appears to be cancer-specific, as earlier mentioned. To date, however, despite a number of studies on EGFR and its downstream signaling pathways, the Arf6 pathway has not been analyzed in HNSCCs. This may be because previous gene expression profiling analyses on clinical samples of HNSCCs and other cancers have not nominated this pathway to be correlated with poor prognosis and/or poor survival, as also earlier mentioned. Protein levels of Arf6 and AMAP1 do not correlate with their mRNA levels in breast cancer cells. Such a lack of correlation might not be specific to breast cancer cells, since we found that mRNAs of both Arf6 and AMAP1 have long 5′-untranslated regions with relatively large free-energy changes and hence are classified as typically 'weak-mRNAs', that are neither immediately nor efficiently translated into proteins on their own [29]. These properties of the *AMAP1* and *Arf6* mRNAs might have hindered them from their previous identification, also in studied on HNSCCs.

In this study, we successfully showed that expression of the AMAP1 protein at high levels, as well as its co-overexpression with EGFR, statistically correlates with poor disease-free survival and poor overall survival of HNSCC patients, while larger numbers of patients need to be analyzed to further generalize this notion. We propose that protein levels of AMAP1, together with protein levels of EGFR, provide a simple and excellent biomarker predictive for the recurrence and survival of HNSCC patients under the current therapeutics. The Arf6 pathway mediates the motile and invasive phenotypes of cancers. These phenotypes are thought to be critical for their resistance to radiotherapy, in which the tumor cells that survive radiation may escape from the sites of radiation, that are hypoxemic and inflammatory due to the radiation. Thus, combinations of AMAP1-targeting treatments and radiotherapy are expected to improve the therapeutic effects against HNSCCs.

## Conclusions

We propose that immunohistochemical staining of the AMAP1 protein on its own, or together with staining of the EGFR protein, provides a simple and reliable diagnosis that predicts the recurrence and survival of HNSCC patients under the current therapeutics. Our identification of these predictive biomarkers, together with our previous findings on the coherent signaling pathway that these biomarkers ultimately generate, namely the EGFR-GEP100-Arf6-AMAP1-cortactin pathway, should be powerful information for the further development of HNSCC therapeutics.

## Materials and methods

### Antibodies

Antibodies against the following proteins were purchased from commercial sources: cortactin (CST, Lake Placid, NY) and EGFR (TDL and 31G7 mAb, Nichirei, Tokyo). Rabbit polyclonal antibodies against AMAP1 and GEP100 were as described previously [21,23]. These rabbit polyclonal antibodies were affinity-purified by use of each corresponding peptide used for the immunization, and their specificity on western blotting and the validity on immunohistochemistry were carefully verified before use [21-24,30].

### Patients and samples

All clinical specimens were selected from patients with primary tongue squamous cell carcinoma, who underwent tongue resection at Hokkaido University Hospital between March 1996 and March 2012, and were analyzed retrospectively. This study was approved by the Institutional Review Board of Hokkaido University Hospital, Japan (study number 012–0166). The requirement for written consent was waived by this board in accordance with the Ethical Guidelines for Clinical Studies of the Japanese Ministry of Health, Labor and Welfare. Clinical stages of the cancers were evaluated according to the 2002 International Union Against Cancer staging system.

### Patient characteristics

The characteristics of and the treatment regimens for the patients (n = 20; 15 men and 5 women) who underwent primary surgical resection for HNSCC in the Departments of Otolaryngology, and Head and Neck Surgery, Hokkaido University Graduate School of Medicine (Sapporo, Japan) are summarized in Table 1. The median age of the patients was 60.7 years (range 31–84 years). The tumor site was the oral cavity (tongue) in all patients. The clinical tumor stage was T2 for 13 patients and T3 for 7 patients. Ten patients were node-positive and 10 were node-negative. Pathological findings revealed that 61.4% (n = 12) of the cancers were classified as well differentiated, 30.7% (n = 7) as moderately differentiated and 7.9% (n = 1) as poorly differentiated. Nine patients (45%) received radiation therapy and three patients (15%) received chemotherapy prior to surgery. The median follow–up for the 12 surviving patients was 65.5 months (range, 5–175 months).

### Immunohistochemistry

The methods of immunohistochemical staining were as described previously [21,23,30]. Briefly, immunohistochemical staining was performed using 3 μm-thick formalin-fixed paraffin-embedded sequential sections as follows. Samples were first deparaffinized in xylene and dehydrated in graded alcohols. After rinsing in TBS buffer (25 mM Tris–HCl (pH 7.4), 137 mM NaCl, and 2.7 mM KCl), the sections were processed for antigen retrieval in a pepsin solution (Nichirei) at 37°C for 10 min (for EGFR) in a 1 mM ethylenedyamine tetra-acetic acid (EDTA) retrieval solution (pH 9.0) (45211 Nichirei), at 95°C for 40 min (for AMAP1 and GEP100). Endogenous peroxidase was then blocked by incubation in 0.3% H_2_O_2_-methanol at room temperature for 10 min. After rinsing with TBS, sections were then incubated with primary antibodies against EGFR (1:50), AMAP1 (1:500) or GEP100 (1:100) for 30 min, then with EnVisionTM (Dako, Tokyo) for 30 min, and finally with peroxidase-conjugated streptavidin (Vector Labs, Burlingame, CA) for 50 min. After rinsing in TBS, the coloring reaction was performed with DAB (Dojin, Kumamoto) for 5 min. Each section was counterstained with hematoxylin. These processes were all performed at room temperature.

### Scoring

Immunohistochemical samples were scored by two investigators, which include one pathologist (K.C.H.). They evaluated the staining of various proteins (EGFR, GEP100, AMAP1, and cortactin) under a light microscope at a magnification of × 200. The staining intensity of each protein in tumor cells was graded on a scale of 0–2, in which no staining was scored as 0, weak staining was scored as 1, and strong staining was scored as 2. Percentages of the stained area were calculated for each specimen, and a proportional score was assigned (0 if 0%, 0.1-0.9 if 10–90%, and 1 if 100%). Proportional scores were multiplied by the staining intensity to obtain a final semi-quantitative H score. Median values of the H score were chosen as the cut off point for distinguishing between the low and high expression levels of each protein [26].

### Statistical analyses

Overall survival was calculated from the date of the surgical treatment to the date of death or the most recent follow-up. Disease-free survival was defined as the time from the date of the surgical treatment to the date of the first observation of disease progression, or relapse, or death due to any cause. Overall survival and disease-free survival were analyzed using the Kaplan-Meier method. The calculated *p*-value was considered to be significant when less than 0.05. These analyses were performed using Statcel® software (OMS Ltd., Tokyo).

## Abbreviations

HNSCCs: Head and neck squamous cell carcinomas; EGFR: Epidermal growth factor receptor; GEF: Guanine nucleotide exchanging factor; PRKD2: Protein kinase D2; TBS: Tries-buffered saline; DFS: Disease-free survival.

## Competing interests

The authors have consigned the antibody against AMAP1 to Gene Science Inc. for its distribution to academic, public usage. This does not alter the authors’ adherence to all the Cell Communication and Signaling policies on sharing data and materials. The authors declare that they have no other competing interests.

## Authors’ contributions

HS, YM, SF, H Sabe, contributed to the design of the study. HS, KCH, YH, H Sabe performed the experiments and analysed the data. HS, YH, HH, AH, H Sabe, compiled the data and prepared a first manuscript. HS, HH, AH, H Sabe, contributed to the writing of manuscript. All authors read and approved the final version of this manuscript.
